# Clinical and Virological Factors Influencing the Performance of a NS1 Antigen-Capture Assay and Potential Use as a Marker of Dengue Disease Severity

**DOI:** 10.1371/journal.pntd.0001244

**Published:** 2011-07-19

**Authors:** Veasna Duong, Sowath Ly, Patrich Lorn Try, Anne Tuiskunen, Sivuth Ong, Norith Chroeung, Ake Lundkvist, Isabelle Leparc-Goffart, Vincent Deubel, Sirenda Vong, Philippe Buchy

**Affiliations:** 1 Institut Pasteur in Cambodia, Réseau International des Instituts Pasteur, Phnom Penh, Cambodia; 2 Paediatric Department, Kampong Cham Provincial Hospital, Kampong Cham, Cambodia; 3 Swedish Center for Infectious Disease Control, Stockholm, Sweden; 4 Unité de Virologie, Institut de Médecine Tropicale du Service de Santé des Armées, Marseille, France; Tropical Medicine Institute Pedro Kourí (IPK), Cuba

## Abstract

**Background:**

Detection of dengue NS1 antigen in acute infection has been proposed for early diagnosis of dengue disease. The aim of this study was to evaluate the clinical and virological factors influencing the performance of the Platelia NS1 Ag kit (BioRad) and to assess the potential use of NS1 antigen and dengue viral loads as markers of dengue disease severity.

**Methodology/Principal Findings:**

Blood specimens were collected from patients hospitalized at the Kampong Cham hospital during the 2006 and 2007 dengue epidemics in Cambodia. Dengue infection was confirmed in 243/339 symptomatic patients and in 17 asymptomatic individuals out of 214 household members tested. Overall sensitivity and specificity of Platelia NS1 Ag kit were 57.5% and 100% respectively. NS1 Ag assay combined with IgM antibody capture ELISA significantly increased the sensitivity for dengue diagnosis. NS1 Ag positivity rate was found significantly higher in DF than in DHF/DSS, in primary than in secondary infections, in patients with a high viremia (>5 log/mL) and in patients infected with DENV-1. In asymptomatic individuals, the NS1 Ag capture sensitivity tends to be lower than that in symptomatic patients. Milder disease severity was observed independently in patients with RNA copy number >5 log10 cDNA equivalents/mL or in high level of NS1 antigen ratio or in DENV-1 infection.

**Conclusions:**

Overall sensitivity of NS1 Ag detection kit varied widely across the various forms of dengue infection or disease. Sensitivity was highest in patients sampled during the first 3 days after onset of fever, in patients with primary infection, DENV-1 infection, with high level of viremia and in DF rather than DHF/DSS. In asymptomatic patients, RT-PCR assay has proved to be more sensitive than NS1 antigen detection. The NS1 antigen level correlated significantly with viremia and a low NS1 antigen ratio was associated with more severe disease.

## Author Summary

Dengue is the most prevalent arthropod-borne disease in tropical regions. The clinical manifestation may vary from asymptomatic to potentially fatal dengue shock syndrome. Early laboratory confirmation of dengue diagnosis is essential since many symptoms are not specific. Dengue non-structural protein 1 (NS1) may be used in simple antigen-capture ELISA for early detection of dengue virus infection. Our result demonstrated that the Platelia NS1 antigen detection kit had a quite low overall sensitivity. However, sensitivity rises significantly when used in combination with MAC-ELISA. When taking into account the various forms of dengue infection, the NS1 antigen detection was found relatively high in patients sampled during the first 3 days of fever onset, in patients with primary infection, DENV-1 infection, with high level of viremia and in mild form of dengue fever. In asymptomatically infected individuals, RT-PCR assay has proved to be more sensitive than NS1 antigen detection. Moreover, the NS1 antigen level correlated significantly with high viremia and low level of NS1 antigen was associated with more severe disease.

## Introduction

Dengue virus (DENV), a mosquito-borne virus (family *Flaviviridae*, genus *Flavivirus*) is an enveloped, single stranded positive-sense RNA virus. There are 4 serologically related but antigenically and genetically distinct dengue viruses (DENV-1, -2, -3, and -4) causing disease in human. While most infections result in asymptomatic response or mild febrile illness (dengue fever or DF), all 4 serotypes are capable of producing the more severe and potentially fatal dengue hemorrhagic fever (DHF) and dengue shock syndrome (DSS) and non-specific complication of systemic diseases (e.g., encephalitis, hepatitis) [1], [2], [3].

With over 2.5 billion people living in area at high risk for infection and an estimated 50–100 million cases of dengue infection every year, DENV has become the most important arthropod-borne virus affecting human [2]. Several factors such as rapid urbanization, failure to control vector mosquitoes and rapid progress in air transportation have contributed to the emergence of endemic dengue in over 100 countries [2], [4].

In Cambodia, there is a high incidence of reported diseases (DF, DHF) of 10,000–12,000 cases annually during 2002–2006 and the case-fatality rate was 1–2% over the past 5 years [5].

Commonly used diagnosis methods are often unable to confirm dengue infection during the acute febrile stage in a timely manner and at a reasonable cost [3], [6]. Virus isolation is a time-consuming and fastidious process that requires specialized laboratory equipments and experienced personnel. The development of reverse transcriptase polymerase chain reaction (RT-PCR) and recently real time RT-PCR techniques have significantly reduced the processing time and permitted the detection of the virus in the early stage of the infection [7]. However, these methods remain expensive and technically difficult, particularly in laboratory settings of the developing world. Serological diagnosis of dengue infection has many advantages including more flexibility, wide availability of reagents, lower cost, and less equipments required [3]. Unfortunately, cross-reactivity between flaviviruses, antibody half-life, need of paired sera, and inability to be detected in acute phase of infection make the serological diagnosis more complicated [3] .

Due to the drawbacks of serological methods to reliably diagnose acute infections, a number of alternative options have been explored [7] and one of the most promising methods was the detection of nonstructural protein 1 (NS1) . NS1, produced in both membrane-associated and secreted forms, may play an essential role in viral replication. The amount of secreted NS1 (sNS1) in the serum of individuals infected with DENV has been shown to directly correlate with viremia and the pathogenesis of dengue infection [8], [9], [10], [11], [12].

The NS1 protein is detectable by enzyme-linked immunosorbent assay (ELISA) as early as the first day of fever and can be found up to 9 days in serum even after RT-PCR detection has become negative [9], [13], [14], [15]. Given all these advantages, NS1-based ELISAs may be an important diagnostic tool for those acute samples in which IgM is not detectable and for which PCR is not available. Thus, NS1 antigen might be useful in early detection and a potential prognosis parameter for severe dengue infections. Several NS1 antigen commercial kits are now available and most of them have been evaluated for their sensitivity and specificity in patients experiencing clinically apparent infections. The sensitivity observed for these assays varied from 63% to 94% [8], [14], [16], [17].

In this study, we evaluated the clinical and virological factors influencing the performance of NS1 antigen-capture assay and assessed the potential association of the level of NS1 antigenemia (using simple semi-quantitative estimation) and the level of viremia with dengue disease severity using well characterized sera from patients presenting at hospitals. NS1 antigen test could be an easy tool to confirm dengue infection in such individuals on a single blood sample during the acute phase of the disease rather than by indirect methods that require at least 2 samples. We also evaluated the test in asymptomatically dengue-infected individuals. Indeed, asymptomatic individuals and unreported patient with mild febrile disease who represent the vast majority of dengue infection are believed to constitute an important reservoir of the virus in countries where dengue is highly endemic [18], [19].

## Materials and Methods

### Patients' recruitment and samples processing

Patients were enrolled in the pediatric ward of the Kampong Cham provincial hospital during 2 consecutive dengue epidemics between May and October in 2006 and 2007. This study was approved by the National Cambodian Ethics Committee and patient's enrolment was subject to obtaining a written consent signed by the patients or the under 16 year old patient's legal representatives.

Patients clinically diagnosed with dengue infection and who fulfilled the following inclusion criteria were enrolled: age ≥≥24 months, fever >38°C and having at least one of the symptoms: rash or severe headache or retro-orbital pain or myalgia or joint pain or bleeding symptom. Patients' information and clinical data were collected by the physicians using a specific case report form and blood samples were taken on hospital admission and discharge. Patients diagnosed for other infections beside dengue and patients hospitalized with a non-infectious disease (e.g., cranial trauma, etc.) were recruited as control group.

Blood samples were tested for haematocrit and platelet count as well as for other biological parameters necessary for patients' follow-up. Sera were tested for dengue using serology and molecular methods at Institut Pasteur - Cambodia.

Patients diagnosed for dengue infections were classified as DF, DHF or DSS using the former WHO criteria [20] as recommended at the time of the study.

In order to identify non-symptomatic cases, family members of dengue-infected patients were visited the next day following laboratory confirmation of the dengue infection (which usually took approximately 24 hours). Their body temperature was followed for 7 days. Blood samples were taken at the first and 7^th^ day of follow-up and if a family member developed fever. A non-symptomatic dengue case was defined as a household member who tested positive for dengue infection but did not display any of the symptoms of the inclusion criteria.

### Laboratory diagnosis

A confirmed dengue infection ("gold standard algorithm") was defined by the detection of anti-dengue virus (DENV)-specific IgM or a 4 fold increase of hemagglutination inhibition (HI) titer in the pair of sera collected with an interval of minimum 7 days and the detection of NS1 antigen in serum by the NS1 Platelia test (BioRad, Hercules, CA) and/or the isolation of DENV after inoculation into mosquito cell lines and/or the detection DENV RNA by RT-PCR or real time RT-PCR assay.

An “in-house” IgM capture Enzyme-Linked Immuno-Sorbent Assay (MAC-ELISA) was used to detect DENV and JEV IgM as the 2 viruses co-circulate in the country [21], [22]. A result was considered positive when the optical density (OD) was > mean OD of three negative control specimens +3 standard deviations. When the anti-JEV result was higher than the anti-DENV result, the subject was not considered to have DENV infection.

HI test was carried out according to the method described by Clarke and Casals [23] adapted to 96-well microtitre plate. Due to the serological cross-reactivity between arboviruses, paired specimens were tested for DENV and JEV hemagglutination-inhibiting antibodies. Primary or secondary acute dengue infection was determined by a fourfold increase in HI titer between the first and second sample according to criteria established by the WHO [20].

The NS1 Platelia antigen detection (BioRad, Marnes-la-Coquette, France) was performed on patient's sera according to the manufacturer's instructions. Samples with equivocal result were repeated and if they were still equivocal they were considered as negative. The optical density (OD) reading obtained with a spectrophotometer at 450/620 nm is proportional to the amount of NS1 antigen present in the sample [13]. The assay provides qualitative and semi-quantitative results in human serum or plasma. The semi-quantitative results were expressed as the ratio calculated by dividing the absorbance measured on the sample by the mean value of the optical densities of 2 cut-off controls. The cut-off value corresponds to the mean value of the OD of the cut-off control provided and tested in duplicate.

The isolation of DENV was performed using mosquito cell line (clone C6/36 of *Ae. Albopictus* cells). Briefly, each acute serum was diluted 1∶20 with L15 Leibovitz Medium (Sigma Aldrich, Steinheim, Germany) in which 2% of fetal calf serum was added. Diluted sera were inoculated into 12-well plate containing 100% confluent C6/36 cells and then incubated for 7 days at 28°C. Cells were harvested, and DENV infection was confirmed by an immunofluorescence assay using dengue serotype-specific monoclonal antibodies as described previously [21], [22].

Viral RNA was extracted from acute phase serum samples using the QIAmp Viral RNA Mini kit (Qiagen, Hilden, Germany). The DENV serotype was determined by RT-PCR based on the technique developed by Lanciotti *et al.* [24] and modified by Reynes *et al.* [25]. The positive samples by conventional RT-PCR were then tested for dengue viral loads by a serotype-specific real-time RT-PCR assay targeting NS5 gene using quantified internal controls [26]. The results were expressed as cDNA equivalents per milliliter of serum. The limit of detection for this assay was 500 cDNA equivalent/mL.

### Statistical analysis

All statistical analyses were performed using Stata/SE version 9.0 (StataCorp, TX, USA). Significance was assigned at *P<*0.05 for all parameters and 95% of interval confidence was used. Categorical variables between groups were compared by Pearson's Chi-squared and Fisher's exact test. T-test and Kruskal-Wallis rank test were used for continuous variables. The correlation coefficients between 2 continuous variables were calculated by Spearman's rank correlation test. For multivariate analyses, we identified independent determinants using a logistic regression model. For clarity, adjustments by the day after onset of fever (DOF) was performed and presented using DOF as a categorical variable: ≤3 days and 4–8 days. In some analyses, DHF and DSS were grouped to increase statistical power.

## Results

A total of 134 and 205 patients were enrolled in 2006 and 2007, respectively, of which 243 patients were diagnosed with acute dengue infection, 62 with non-dengue infection and 17 as having non infectious disease. The summary of patient's characteristics, clinical and virological data of this study is shown in Table 1.

**Table 1:** Summary of demographic, clinical and virological information of studied population.

Variables	Years		
	2006	2007	Total
Acute dengueSymptomatic Asymptomatic	107#90/117 (77%)17/214 (8%)	153#153/205 (75.5%)0	260#243/322 (75.5%)17/214 (8%)
Non-dengue infection	27	35	62
Non infectious disease	0	17	17
Age (median, iqr*)	8 (5–11)	6 (4–8)	7 (4–9)
Sex (female, %)	50 (46.7%)	86 (56%)	136 (52.3%)
Median of day of illness (range)	4 (2–6)	5 (1–8)	4 (1–8)
**Dengue diagnosis (n = 260)**			
Virus isolation	48 (45%)	46 (30%)	94 (36%)
RT-PCR	91 (85%)	110 (72%)	201 (77%)
NS1 antigen assay	73 (68.2%)	77 (50.3%)	150 (57.7%)
MAC-ELISA Positive in acute serumSeroconversionNegative	97 (90.6%) 21 (19.5%)76 (71%)10 (9.4%)	148 (96.7%)92 (60%)56 (36.7%)5 (3.3%)	245 (94.2%)113 (43.5%)132 (50.7%)15 (5.8%)
Hemagglutination-Inhibition assay (titer)Fourfold rise in antibodies on pair seraNo change or less than fourfold riseData not available¥	87 (81.3%)7 (6.5%)13 (12.2%)	134 (87.6%)7 (4.6%)12 (7.8%)	221 (85%)14 (5.4%)25 (9.6%)
**DENV serotypes**			
DENV-1	40 (37%)	15 (10%)	55 (21%)
DENV-2	2 (2%)	8 (5%)	10 (4%)
DENV-3	47 (44%)	74 (48.5%)	121 (46.5%)
DENV-4	2 (2 %)	13 (8.5 %)	15 (5.8%)
Unknown serotype	16 (15%)	43 (28%)	59 (22.7%)
**Clinical manifestation**			
DF	73 (68%)	28 (18%)	101 (39%)
DHF	17 (16%)	25 (16%)	42 (16%)
DSS	0	45 (29.5%)	45 (17%)
Indeterminate clinical status	17 (16%)	55 (36%)	72 (28%)
**Serological status**			
Primary	24/87 (28%)	8/134 (6%)	32/221 (14.5%)
Secondary	63/87 (72%)	126/134 (94%)	189/221 (85.5%)
Indeterminate or unknown	20	19	39

*interquartile range.

# Numbers used as denominator for each column, otherwise indicated.

**¥:** Insufficient serum volume or no second serum.

Using the former WHO criteria [20], 101 dengue patients were classified as DF, 42 as DHF, 45 as dengue with DSS and 72 as indeterminate. The inability to classify these 72 patients was due to the lack of clinical and laboratory data necessary for the classification or they did not meet all the four WHO criteria (see materials and methods). After measuring HI titers on paired sera, dengue cases were classified in primary infections (n = 32, 14.5%), secondary infections (n = 189, 85.5%) but in 39 cases it was not possible to determine the immune status.

During the household investigation, 17 (8%) dengue-infected individuals who did not experience any symptoms and 2 (1%) symptomatic household members of 15 dengue index cases (DIC) were identified among 214 household members (Table S1).

**Figure 1:**
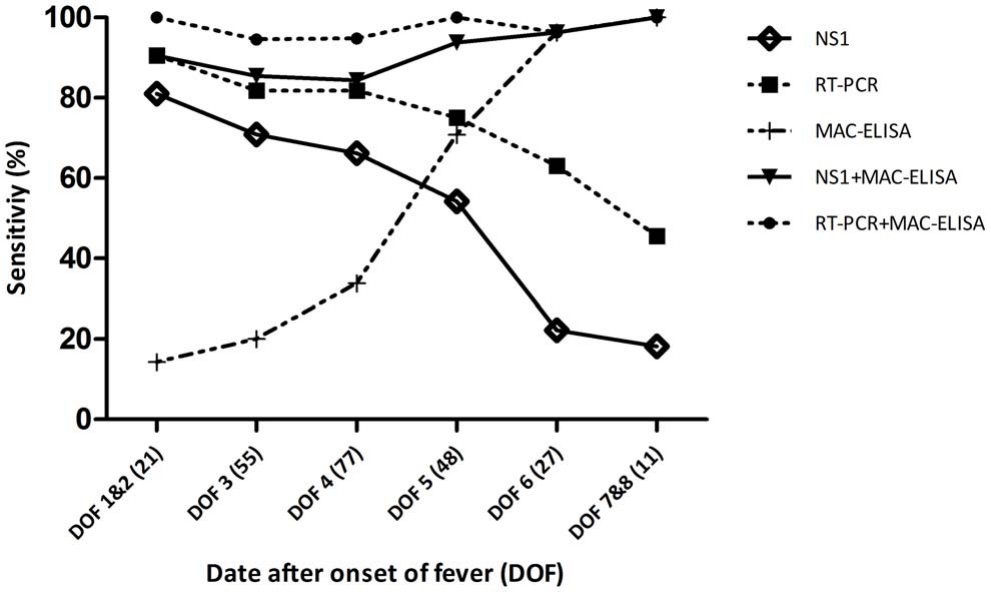
Sensitivity of Platelia NS1, MAC-ELISA and RT-PCR depending on DOF* (n = 239). *DOF: Day after onset of fever.

Figure 1 shows the overall positive rate of NS1-antigen capture assay, RT-PCR and MAC-ELISA in relationship with the day after onset of fever. During the first 2 days in the course of the disease the sensitivity of NS1 assay and RT-PCR reached the highest values (81% and 90.5%, respectively) and then decreased to less than 20% and 45.5% respectively by day 7–8. On the contrary, the number of specimens positive by MAC-ELISA increased steadily from less than 20% at day 1–2 to 100% by day 7.

The sensitivity and specificity of the NS1-capture assay were 57.7% (95% CI: 51.4–63.8%) and 100% respectively (Table 2). When diagnosed solely by RT-PCR, the sensitivity was 77.3% (95% CI: 71.7–82.2) and the specificity 100%. When samples were collected during the first 3 days of illness, the sensitivity of the NS1 commercial test improved: 74% (95% CI: 62.8–83.4) in the dengue-confirmed case group and 82.3% (95% CI: 69.5–90.0) in the group of individuals who tested positive by RT-PCR only.

**Table 2:** Sensitivity, specificity, positive and negative predictive values of Platelia NS1 assay against dengue-confirmed cases.

	Studied population	Acute dengue infection	NS1 positive	Sensitivity % [CI95%]	Specificity % [CI95%]	PPV % [CI95%]	NPV % [CI95%]	*p* value$
Total	339*	260#	150‡	57.7 [51.4–63.8]	100	100	41.8 [34.7–49.2]	*p*<0.001
DOF 1–3	110	77	57	74 [62.8–83.4]	100	100	62.3 [47.9–75.2]	*p*<0.001
DOF 4–8	196	163	85	52.2 [44.2–60.0]	100	100	29.7 [21.4–39.1]	*p*<0.001

*33 cases with imprecise DOF were excluded.

# 20 cases with imprecise DOF were excluded.

**‡:** 8 cases with imprecise DOF were excluded.

$ *P* values refer to 2×2 contingency comparison between % of NS1 positive cases and % of dengue confirmed cases.

PPV: positive predictive value.

NPV: negative predictive value.

The NS1 antigen kit combined with MAC-ELISA detected a significantly higher number of acute dengue cases than NS1 antigen kit alone (overall sensitivity: 85.7% vs. 57.7%; *p*<0.001, Table S2). An increased sensitivity was also observed when combining RT-PCR and MAC-ELISA results (overall sensitivity: 95.4% vs. 77.3% for RT-PCR alone, *p*<0.001, Table S2). The comparison with DOF subgroups was detailed in Table S2.

**Figure 2:**
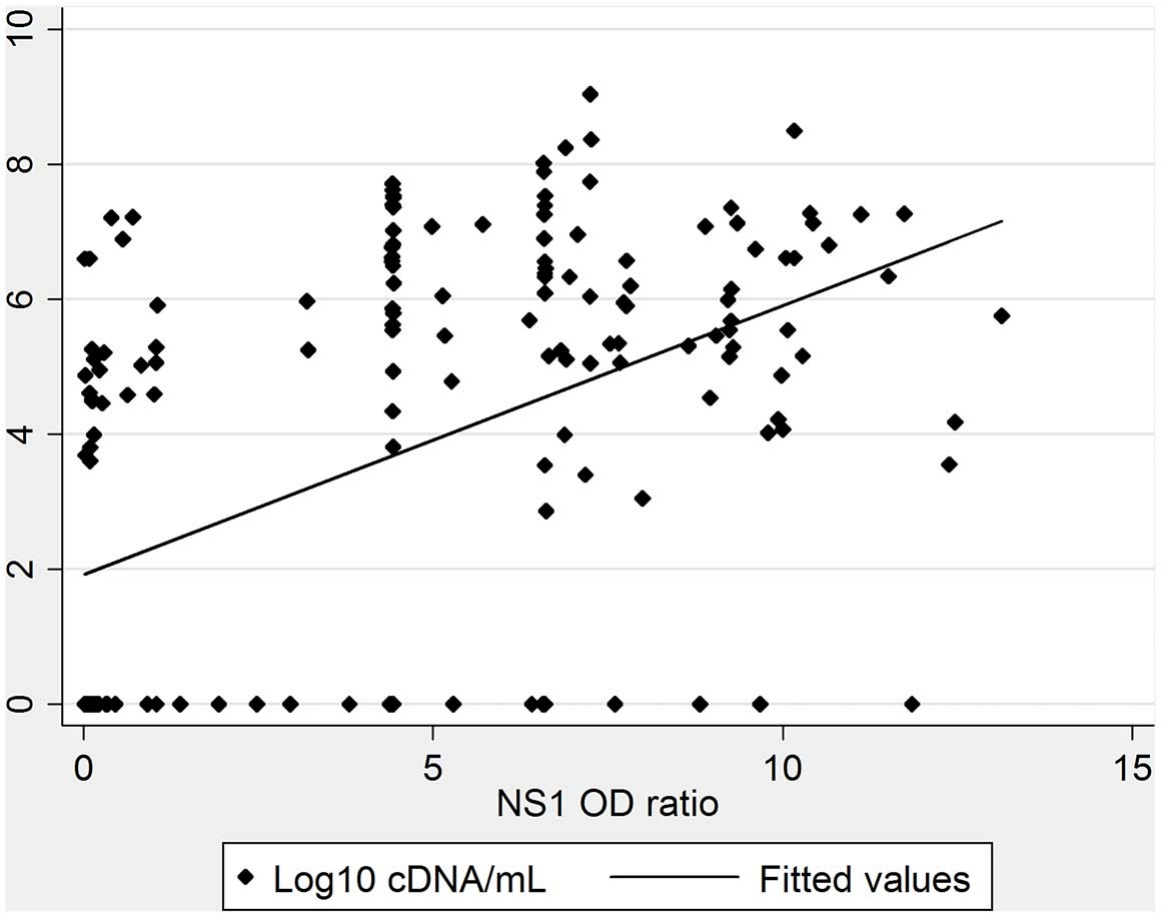
Comparison of NS1 antigen OD* ratio and viral load (log 10 cDNA equivalents/mL). *OD: optical density.

When analyzing all dengue-infected individuals, the NS1 antigen ratio correlated with the RNA load of cDNA equivalents per milliliter (r = 0.540, *p*<0.001; Fig. 2).

The overall sensitivity of the NS1 detection kit was significantly higher in DF (72.3%; 95% CI: 63.5–81%) than in DHF/DSS (40.2; 95% CI: 29.85–51.3). But the difference in NS1-capture assay sensitivity was significant only for samples collected after day 3 of fever and not for specimen obtained during the very early phase of the disease (Table 3).

**Table 3:** Sensitivity of Platelia NS1 assay in DFand DHF/DSS patients according to timing of sample collection after DOF*.

	No. of sera tested	NS1 positive	DF (n = 101)		DHF/DSS (n = 87)		*p* value$
			No. of Positive/total tested	Sensitivity % [95% CI]	No. of positive/total tested	Sensitivity % [95% CI]	
**Total**	188¤	108#	73/101	72.3 [63.5–81]	35/87	40.23 [29.85–51.3]	*p*<0.001
**DOF 1**–**3**	55	43	31/41	75.6 [59.7–87.6]	12 /14	85.7 [57.2–98.2]	*p* = 0.407
**DOF 4**–**8**	119	60	38/49	77.5 [63.4–88.2]	22/70	31.4 [20.8–43.6]	*p*<0.001

*DOF: Day after onset of fever.

**¤:** 14 cases with imprecise DOF were excluded.

# 5 cases with imprecise DOF were excluded.

$ *P* values refer to the comparison between NS1 positive rate of DF vs. DHF/DSS for total and for each DOF subgroup.

CI: confidence interval.

The sensitivity of the NS1-capture assay was significantly higher in primary dengue infection (87.5%; 95% CI: 70.0–96.5) than in secondary infection (53.5%; 95% CI: 46.1–60.7) (*p*<0.001). The difference was also significant in the DOF 4–8 group (*p* = 0.002) and at the limit of significance in the DOF 1–3 group (*p = *0.055).

The sensitivity of the test also varied with the virus serotype. It was significantly higher in DENV-1-infected patients (80%; 95% CI: 67–89.6) than in DENV-2- (40%; 95% CI: 12.2–73.8, *p = *0.008), DENV-3- (63.6%; 95% CI: 54.4–72.2, *p = *0.03) and DENV-4- (53.3%; 95% CI: 26.6–78.7, *p* = 0.03) infected patients although the difference was only significant for the DOF 4–8 group (Table 4).

**Table 4:** Sensitivity of NS1 assay for each DENV serotype.

	DENV-1		DENV-2		DENV-3		DENV-4		*p* value$
	NS1 positive/total tested#	Sensitivity% [95% CI]	NS1 positive/total tested	Sensitivity% [95% CI]	NS1 positive/total tested‡	Sensitivity% [95% CI]	NS1 positive/total tested	Sensitivity% [95% CI]	
**Total**	44/55	80.0 [67.0–89.6]	4/10	40.0 [12.2–73.8]	77/121	63.6 [54.4–72.2]	8/15	53.3 [26.6–78.7]	*p*<0.05
**Day 1**–**3**	21/25	84 [63.9–95.5]	2/3	66.6 [9.4–99.2]	27/32	84.3 [67.2–94.7]	3/5	60.0 [14.7–94.7]	*p*>0.05
**Day 4**–**8**	21/25	84.0 [63.9–95.5]	2/7	28.6 [3.7–70.9]	48/79	60.7 [49.1 –71.6]	5/10	50.0 [18.7–81.3]	*p*<0.05

# 5 cases with imprecise DOF were excluded.

**‡:** 10 cases with imprecise DOF were excluded.

$ *P* values refer to comparison between NS1 positive cases in DENV-1 and DENV-2, DENV-3 or DENV-4 groups.

CI: confidence interval.

DF was significantly more frequent after infection with DENV-1, compared to other dengue virus infections (38/47, [80.85%, 95% CI: 66.7–90.8] vs. 4/8 [50%,95% CI: 15.7–84.3] for DENV-2, 42/89 [47.2%, 95% CI: 36.5–58] for DENV-3 and 5/9 [55.6%, 95% CI: 21.2–86.3] for DENV-4; *p* = 0.002). The proportion of primary infections in DENV-1-infected patients was 20% versus 12.3% for the overall studied population (*p = *0.13).

Of 201 RT-PCR positive samples, 189 were tested for RNA quantification using real-time RT-PCR. Among these, 70 samples were containing RNA levels lower than the detection limit of the real time RT-PCR. During clinical classification, 44 cases were excluded from the analysis because the clinical information was not sufficiently precise to allow a classification according the WHO criteria. NS1 antigen-capture assay's sensitivity was significantly higher in patients with viremia >5 log cDNA equivalents/mL than <5 log cDNA equivalents/mL regardless the clinical severity and day of sample collection after onset of fever (Table 5). The overall NS1 antigen-capture sensitivity in patients with viremia >5 log cDNA equivalents/mL was 91% versus 45% in patients with viremia <5 log equivalents/mL (*p<*0.001).

**Table 5:** Sensitivity of NS1 test compared with level of viral RNA in serum (log10 cDNA equivalents/mL).

	DF (n = 88*)NS1 positive/total tested [sensitivity; 95% CI]			DHF/DSS (n = 57#)NS1 positive/total tested [sensitivity; 95% CI]		
	<5 log/ml	>5 log /ml	*P* value	<5 log/ml	>5 log /ml	*p* value
Total	19/33 [57.6%; 39.2–74.5]	51/55 [92.7%; 82.4–98]	*p*<0.001	14/41 [34% ; 20.1–50.6]	15/16 [93.8%; 69.8–99.8]	*p*<0.001
DOF 1–3	7/11 [63.6%; 30.8–89.1]	24/26 [92.3%; 74.8–99]	*p* = 0.035	1/2[50%; 1.2–98.7]	10/10 [100%; 69.2–100]	*p*>0.05
DOF 4–8	12/17 [70.6%; 44–89.7]	24/25 [96%; 79.6–99.9]	*p* = 0.021	13/38 [34.2%; 19.6–51.4]	5/5 [100%; 47.8–100]	*p* = 0.009

*9 cases with imprecise DOF were excluded.

# 2 cases with imprecise DOF were excluded.

CI: confidence interval.

In asymptomatic individuals the sensitivity of NS1 test was significantly lower than that in DIC (35.3% versus 86.7%, *p* = 0.003; Table S1) and at the limit of significance when compared to the sensitivity observed in all symptomatic cases (59.3%, *p = *0.053). Seventy three percent (8/11) of the asymptomatic individuals experienced secondary infection which was lower than in DIC (100%, *p = *0.063). The levels of viremia expressed in log10 cDNA equivalents/mL in asymptomatic individuals was significantly lower than in DIC (2.72, SD: 2.72, n = 13 vs. 4.96, SD: 2.37, n = 15; *p = *0.043) but the difference was not significant if compared with the level of viremia in all dengue confirmed cases (3.79, SD: 3.06, n = 176; *p = *0.145).

In these asymptomatic individuals, nested RT-PCR detection was significantly more sensitive than NS1 antigen-capture assay (76.5% vs. 35.3%, *p = *0.015).

**Figure 3:**
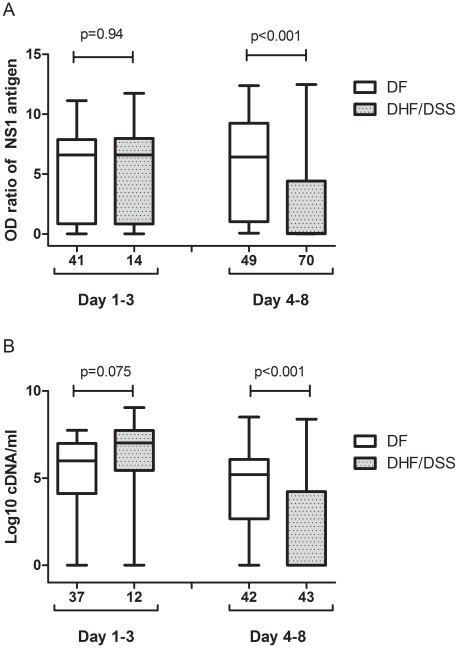
Level of NS1 antigen and viremia by disease severity. Shown are the median, interquartile and 95 percent range of OD ratio of NS1 antigen (A) and log10 cDNA equivalents/mL (B) distributed by disease severity (DF, DHF/DSS). The number of patients is shown under the X axis bar. NS1 antigen OD ratio was significantly higher in DF group than in DHF/DSS group (*p*<0.001) at DOF 4–8 (A). Log10 cDNA equivalents/ml was significantly higher in DF group than in DHF/DSS group at DOF 4–8 (*p*<0.001) (B).

In multivariate analysis, DHF/DSS were independently associated with secondary infection (adjusted OR = 6.6, *p = *0.01) when controlled with age, day of fever onset, DENV serotypes and immunity status (primary/secondary infection). Out of 77 DHF/DSS patients, 74 (96%) had secondary dengue infection. Milder disease severity was associated with high NS1 antigen level (adjusted OR: 0.21, *p = *0.002) (Table S3A and Fig. 3A) or DENV-1 infection (adjusted OR: 0.083, *p = *0.006). Similar results were found in multivariate analysis when using the number of cDNA copies instead of NS1 antigen OD ratio: association persisted between DHF/DSS and secondary infection (adjusted OR = 6.03, *p = *0.01) and milder disease severity was observed in patients with cDNA copy number >5 log10 cDNA equivalents/mL (adjusted OR = 0.33, *p = *0.019) (Table S3B and Fig. 3B).

## Discussion

DENV NS1 antigen is detected in the blood circulation as early as viral RNA [8], [9], [12], [13]. Thus its detection is useful for early dengue diagnosis and could be used as an easy, fast and feasible alternative to RT-PCR in developing countries. For this reason, the sensitivity of a commercial NS1 antigen detection kit was studied in context of several factors: severity of the infection including asymptomatic dengue-infected individuals, time of sampling, serological status (primary or secondary infection), DENV serotype and level of viremia in acute sample.

The overall sensitivity of Platelia Dengue NS1 Ag kit (58%) is slightly lower than that observed in previous studies (63–94%) [8], [14], [16], [27], [28], although, the excellent specificity reported here is in agreement with results provided by other authors (98.4–100%) [8], [14], [16], [27], [28]. The sensitivity of the test was better during the early stage of the illness (before day 4). The modest overall sensitivity reported here was comparable to that of a recent multi-country NS1 antigen assay evaluation [28] which showed a 66% (range: 34% to 76%) sensitivity of the NS1 antigen detection by Platelia kit. The relatively low sensitivity NS1 antigen detection in the current study is probably due to the high number of secondary infections (85.5%) which reflects of the true situation in Cambodia and other dengue hyper-endemic countries [14], [29], [30]. Indeed, we recorded a lower sensitivity of the NS1 antigen-capture assay in secondary infections in comparison to primary infections (87.5% vs. 53.5%). Anti-NS1 antibodies are more frequently detected in dengue secondary infection [31] and the antibody-antigen complex impedes the test's ability to detect free NS1 [9], [12]. A dissociation of NS1 antibody-antigen immune complexes would increase the sensitivity of NS1 antigen detection [31] but such a method is unfortunately not offered in the commercial kits and was not performed in our study.

When the NS1 antigen assay was coupled with MAC-ELISA, the overall sensitivity increased by 28%. This combination of NS1-antigen capture assay and IgM antibody detection for dengue diagnosis showed higher sensitivity than RT-PCR alone and a slightly lower sensitivity than RT-PCR combined with IgM antibody detection. When performed together, NS1 antigen-capture and IgM assays appear to be highly sensitive and complementary, allowing a sufficiently good presumptive (IgM) or definitive (NS1) diagnosis during the acute and the convalescent phase of the disease. This advantage of the combination was positively demonstrated in the multi-country study by Guzman et al. [28]. Moreover, both assays are easy to perform, fast, require limited equipments and expertise, and are affordable. The combination of NS1 antigen and dengue IgM/IgG used in rapid diagnostic test (RDT) format for dengue infection detection has shown in previous studies to be more sensitive than NS1 antigen detection alone and can be used as a “point of care” diagnosis [30], [32].

The sensitivity of NS1 antigen-capture assay was significantly higher for DENV-1 than for the three other serotypes. This could be explained by the higher level of viremia measured during DENV-1 infection than that in patients infected with any of the three other DENV serotypes, although the difference was significant only when DENV-1 was compared with DENV-3 (data not shown). Other factors like a better affinity of the NS1 probe and monoclonal antibodies used in the assay for the DENV-1 strains circulating in Cambodia or the variations in the performances of the RT-PCR method used to establish the diagnosis [28] could also explain this observation. A multi-country evaluation of NS1 antigen capture assay has shown that the sensitivity was highest in DENV-1 infection and lowest in DENV-2 [28]. Additionally, two Vietnamese studies has also shown that the sensitivity in DENV-1 infection was significantly higher than in DENV-2 but not in DENV-3 infected patients and data on DENV-4 was not available [8], [33]. The lowest sensitivity of NS1 antigen capture assay observed in DENV-2 infections (40%) - particularly at DOF 4–8 - might only be a bias due to the limited number of DENV-2 cases included in our study.

Unlike other self limited viral diseases, dengue infection may develop into the life threatening DSS form in a few days. A test allowing early diagnosis, which can predict a risk of subsequent evolution to the severe form is desirable in order to improve the clinical management of dengue infection. This could reduce unnecessary use of antibiotics, hospitalization of patients with milder disease in countries with limited resources and allow early hospitalization and supportive care of those developing potentially life threatening DHF. Quantification of viremia by real-time RT-PCR methods might be useful in this regard [9], [10] but is expensive and not readily available in endemic regions, which hampers its use in clinical practice.

This present study demonstrates a moderate correlation of the semi-quantitative result of NS1 antigen-capture assay with the level of viremia quantified by real time RT-PCR. This finding is in agreement with previous studies in which NS1 antigen-capture assay was demonstrated *in vitro* to be applicable as an easy and fast method for semi-quantification of DENV in cell culture [12], [13], [34] and NS1 levels were found *in vivo* to correlate with viremia level [8], [9]. However, as also stated by Ludert et al. [34], a limitation of the use of Platelia NS1 antigen capture kit as a semi-quantitative test was that we did not use quantified NS1 protein as internal control and our sera were not serially diluted.

As expected and already largely described, DHF/DSS cases are more frequently observed in secondary infection with an adjusted odd ratio of 6.6 [10], [31], [35], [36]. The apparent lowest severity of DENV-1 infections observed in our study is partially in agreement with data published by Vaughn et al. [10] who reported that this serotype caused less severe pleural effusion than DENV-2 but not than DENV-3 and DENV-4 secondary infections. Due to the low number of DENV-2 and also DENV-4 cases recruited in our study but also at the country level (with DENV-2 representing 9.2% and 9.1% and DENV-4 accounting for 2.9% and 3.1% of the serotypes isolated out of the 16,635 and 39,618 dengue cases reported in 2006 and 2007, respectively) [5], we cannot discuss it further.

Interestingly, the mildest dengue infection was also associated with high NS1 antigen level semi-quantitatively measured by the Platelia Dengue NS1 Ag kit (OR = 0.21, *p = *0.002) and in patients infected with DENV-1 (OR = 0.083, *p = *0.006). These findings contrasted with those of studies which showed conversely that a higher viremia titer [10] and NS1 plasma levels were associated with more severe disease [9]. Of note, these studies were conducted on fewer cases and measured the viremia in patients recruited less than 72 hours after fever onset while our results were based on more patients, although only 14/84 DHF/DSS cases were included before DOF 4, and we included additional characteristics that were controlled in the multivariate analysis (i.e. patient's age, day of sample collection after fever onset, DENV serotypes and anti-DENV immune status with well characterized clinical and biological data from hospitalized patients). In addition, in our series, all ambiguous data in regard to severity or primary/secondary dengue infection classification were excluded. The multivariate analysis in principle would avoid the confounding factor introduced by the higher proportion of DENV-1 infections, associated with higher NS1 titers, observed among mild DF cases. Nonetheless, in a multi-country study, Guzman et al. did not find any association between the NS1 detection and disease severity [28]. Since our study was the first to find this association and considering the limitations in the use of Platelia kit for a semi-quantification of NS1 antigen, a more explicit study will be needed to confirm our results.

The same multivariate analysis but using viral load found that patients with viremia lower than 5 log10 cDNA equivalents/mL experienced more severe dengue infection (28% vs. 62.5%, adjusted OR = 0.33, *p = *0.019). The result suggests that low level of viral load and NS1 antigen increases the likelihood of developing severe dengue infection at least in the context of Cambodian DENV strains in circulation and/or population enrolled during the period of this study. The enhanced anti-DENV immune response associated with the severity of the disease [37] and leading to an increased infected cell mass at the early stages of the disease may afterwards accelerate the virus clearance from the serum. Since the Platelia NS1 assay's sensitivity is enhanced after immune complexes dissociation [14], [31], the lower antigenemia or viremia observed in the severe cases could be the result of a higher anti-NS1 immune response.

Indeed, it has already been suggested that the sNS1 can be trapped within immune complexes which impedes the detection by the antigen-capture assays by preventing the plate-bound or probe monoclonal antibodies to access the NS1 target epitopes [31]. Koraka et al. have shown that the dissociation of NS1 antigen-antibody immune complexes improved the sensitivity of their in-house test, particularly in sera collected during secondary infections [31]. Lapphra et al. observed an increase of sensitivity of the Platelia kit by 10% using acid treatment [14]. Unfortunately, immune complex dissociation was not performed in our study to confirm these findings on Cambodian samples.

Immune complex formation with sNS1 [12], [38] and sNS1 binding to endothelial cells [39] have been proposed as potential factors in DHF pathogenesis. In addition, antibodies directed against NS1 cross-react with human platelets and endothelial cells [40]. Anti-NS1 antibodies induce endothelial cells to undergo apoptosis and *in vitro* experiments demonstrated that these antibodies were responsible for an increased endothelial cell monolayer permeability [40]. NS1 may also activate complement by alternative pathway and this might explain the complement activation observed in infants with DHF during primary infections [41].

Our two former hypotheses in their attempts to explain the low antigenemia and viremia observed in the severe cases do not take into consideration the role of the virus. Some strains might be more virulent than others and we cannot rule out the possibility that a lower antigenemia and viremia could be at least partially also the consequence of a lower virus replication. Indeed, our observation is supported by *in vitro* experiments conducted by Tuiskunen et al (personal communication) using the DENV strains collected in this study during the same epidemic year. The *in vitro* study demonstrated that DENV-1 strain isolated from severe dengue infection (DSS) had lower level of replication in mammalian Vero cells than strains isolated from DF and DHF patients. Nonetheless, a non significant relationship between disease severity and level of NS1 antigen or DENV serotype detected in patients was reported elsewhere [8], [28], Hence, our findings might be relevant only for the DENV-1 strains circulating in Cambodia.

Another interesting aspect of this study was the recruitment of individuals asymptomatically infected. In this group, NS1 antigen-capture assay was significantly less sensitive than in the DIC (*p = *0.003). The lower sensitivity of NS1 antigen-capture in asymptomatic patients might be explained by the lower level of viremia in these individuals (Table S1). Along the same line, RT-PCR detection was more sensitive than NS1 antigen-capture assay in detecting infection in apparently healthy individuals (76.5% vs. 35.3% respectively, *p = *0.01) but the difference was not significant in DIC. The level of viremia in asymptomatic cases was not significantly lower than in all dengue confirmed cases (*p = *0.145). Since asymptomatic individuals did not experience more primary infections than DIC, this observation is probably not related to the presence of more anti-NS1 antibodies in one group rather in the other. In addition, the positivity rate of RT-PCR or real time RT-PCR was not significantly different between the two groups. Additional evaluations using greater numbers of asymptomatic cases would probably be helpful to address more explicitly the question of the mechanism of the lower sensitivity of NS1 antigen capture test in this particular group.

In conclusion, we have shown the usefulness of qualitative result of NS1 antigen detection assay in early recognition of dengue infection particularly in combination with IgM test. The point of care rapid diagnostic tests including NS1 antigen and IgM/IgG detection would be probably a helpful tool for early dengue infection diagnosis in clinical practice but these tests need to be further extensively evaluated.

The evaluation of the Platelia NS1 Ag detection kit exhibited a quite low overall sensitivity. These data suggest that the NS1 antigen results should be interpreted with caution when used alone. However, its sensitivity was relatively high in patients who were sampled during the first 3 days after the onset of fever, in patients with primary infection, in patients with DENV-1 infection, in patients experiencing a high level of viremia and in patients with dengue fever forms. In asymptomatic patients, RT-PCR assay has proved to be more sensitive than NS1 antigen detection.

Moreover, using the semi-quantitative approach of the test, we have demonstrated that the NS1 antigen level was significantly correlated to the level of viremia and that the low level of NS1 antigen was associated with more severe disease.

## Supporting Information

Table S1 **Virological results and clinical features of dengue index cases and household members (in 74 households).**
(DOC)

Table S2 **Comparison of NS1 kit or RT-PCR sensitivity against the combination of each assay with MAC-ELISA.**
(DOC)

Table S3 A. Multivariate analysis of factors* associated with DHF/DSS. B. Multivariate analysis of factors* associated with DHF/DSS.
(DOC)

Checklist S1 **STARD checklist.**
(DOC)
