# Supplementary material for: Clinical and Virological Factors Influencing the Performance of a NS1 Antigen-Capture Assay and Potential Use as a Marker of Dengue Disease Severity
Source: PLoS Negl Trop Dis. 2011 Jul 19;5(7):e1244. doi: 10.1371/journal.pntd.0001244 (PMC3139664; doi:10.1371/journal.pntd.0001244)
Supplement: Table S1 — Virological results and clinical features of dengue index cases and household members (in 74 households). (DOC) [file pntd.0001244.s001.doc]

**Table S1. Virological results and clinical features of dengue index cases and household members (in 74 households).**

|  | Household members (n=214) | | Dengue index cases | *p* value* |
| --- | --- | --- | --- | --- |
|  | Presence of symptoms | Absence of symptom |  |  |
| Total of positive cases | 2 (1%) | 17^#^ (8%) | 15 |  |
| NS1 antigen capture assay | 2 (100%) | 6/17 (35.3%) | 13/15 (86.7%) | *p=*0.003 |
| RT-PCR positive  DENV serotype  DENV-1  DENV-3 | 2 (100%)  2  - | 13/17 (76.5%)  4 (30.8%)  9 (69.2%) | 14/15 (93.3%)  7 (50%)  7 (50%) | *p=*0.338 |
| qRT-PCR  Positive  Mean viremia (log cDNA equivalent/mL) | 2/2  5.6 (SD: 0.43) | 7/13 (54%)  2.7 (SD: 2.7) | 12/14 (85.7%)  4.96 (SD: 2.37) | *p=*0.103  *p=*0.043 |
| IgM  Positive (acute serum)  Seroconversion  Negative (on pair sera)^$^ | 2  -  - | 1/17 (6%)  9/17 (53%)  3/17 (41%) | 1/15 (6.7%)  14/15 (93.3%)  - | *p=*0.011 |
| **Clinical manifestation**  DF  DHF/DSS  Indeterminate clinical status  **Serological status**  Primary  Secondary  Unknown | 2  -  -  -  -  2 | -  -  -  3/11 (27%)  8/11 (73%)  6 | 12/15 (80%)  2/15 (13.3%)  1/15 (6.7%)  -  15/15 (100%)  - | *p=*0.063 |

SD: standard deviation

DENV: dengue virus

qRT-PCR: quantitative RT-PCR

N/A: not available

^#^ 2 households had 2 asymptomatic individuals

* *P* value for comparison between asymptomatic individual and dengue index case group

^$^ Second serum was not available in 4 cases
