# Supplementary material for: Clinical and Virological Factors Influencing the Performance of a NS1 Antigen-Capture Assay and Potential Use as a Marker of Dengue Disease Severity
Source: PLoS Negl Trop Dis. 2011 Jul 19;5(7):e1244. doi: 10.1371/journal.pntd.0001244 (PMC3139664; doi:10.1371/journal.pntd.0001244)
Supplement: Table S2 — Comparison of NS1 kit or RT-PCR sensitivity against the combination of each assay with MAC-ELISA. (DOC) [file pntd.0001244.s002.doc]

**Table S2. Comparison of NS1 kit or RT-PCR sensitivity against the combination of each assay with MAC-ELISA.**

| **NS1 antigen capture assay** | | | | **RT-PCR** | | | |
| --- | --- | --- | --- | --- | --- | --- | --- |
|  | NS1 positive  [sensitivity , 95%CI] | NS1 + IgM positive  [sensitivity , 95%CI] | *p* value |  | RT-PCR positive  [sensitivity , 95%CI] | RT-PCR + IgM positive  [sensitivity , 95%CI] | *p* value |
| Total (n=260)^#^ | 150  [57.7%, 51.4-63.8 ] | 223*  [85.7%, 80.9-89.8] | *p*<0.001 | Total (n=260)^#^ | 201  [77%, 71.7-81.2] | 248*  [95.4%, 92.1-97.6] | *p*<0.001 |
| DOF 1-3 (n=77) | 57  [74%, 62.8-83.4] | 67^¥^  [87%, 77..4-93.6] | *p*=0.042 | DOF 1-3 (n=77) | 65  [84.4%, 74.5-91.7] | 74^¥^  [96%, 89-99.2] | *p*=0.014 |
| DOF 4-8 (n=163) | 85  [52%, 44.2-60] | 147*  [90.2%,84.5-94.3] | *p*<0.001 | DOF 4-8 (n=163) | 121  [74%, 66.8-80.7] | 158*  [97%, 93-99] | *p*<0.001 |

^#^ 20 cases with imprecise DOF were excluded.

* Significant difference (*p*<0.001) for comparison between NS1+IgM positive and RT-PCR+IgM positive groups

^¥^ No significant different (*p*=0.35) for comparison between NS1+IgM positive and RT-PCR+IgM positive groups
