# Supplementary material for: Clinical and Virological Factors Influencing the Performance of a NS1 Antigen-Capture Assay and Potential Use as a Marker of Dengue Disease Severity
Source: PLoS Negl Trop Dis. 2011 Jul 19;5(7):e1244. doi: 10.1371/journal.pntd.0001244 (PMC3139664; doi:10.1371/journal.pntd.0001244)
Supplement: Table S3 — A. Multivariate analysis of factors* associated with DHF/DSS. B. Multivariate analysis of factors* associated with DHF/DSS. (DOC) [file pntd.0001244.s003.doc]

**Table S3: Multivariate analysis of factors* associated with DHF/DSS using A) NS1 OD ration and B) Log10 cDNA.**

|  | **Disease severity**  Number of positive/number tested (%) | | **Logistic regression** | | |
| --- | --- | --- | --- | --- | --- |
|  | **DF** | **DHF/DSS** | **Adjusted Odds Ratio** | **95% CI** | ***P* value** |
| Age (number, mean, SD) | 111, 8.72, 9.74 | 87, 7.42, 3.75 | 1.087 | 0.947-0.237 | 0.237 |
| DOF 1-3 | 41/90 (45.5) | 14/84 (16.7) | 1 | - | - |
| DOF 4-8 | 49/90 (54.5) | 70/84 (83.3) | 2.408 | 0.959-0.061 | 0.061 |
| NS1 group 1 (OD ratio <1) | 28/101 (27.7) | 55/87 (63.2) | 1 | - | - |
| NS1 group 2 (OD ratio 1-6) | 20/101 (19.8) | 14/87 (16.1) | 0.363 | 0.121-0.07 | 0.070 |
| **NS1 group 3 (OD ratio > 6)** | **53/101 (52.5)** | **18/87 (20.7)** | **0.214** | **0.08-0.002** | **0.002** |
| RT-PCR negative | 12/101 (12) | 23/87 (26.5) | 1 | - | - |
| **DENV-1** | **38/101 (37.6)** | **9/87 (10.3)** | **0.083** | **0.014-0.006** | **0.006** |
| DENV-2 | 4/101 (4) | 4/87 (4.6) | 0.124 | 0.014-0.064 | 0.064 |
| DENV-3 | 42/101 (41.6) | 47/87 (54) | 0.368 | 0.077-0.212 | 0.212 |
| DENV-4 | 5/101 (5) | 4/87 (4.6) | 0.203 | 0.026-0.126 | 0.126 |
| Primary infection | 26/86 (30.2%) | 3/77 (3.9%) | 1 | - | - |
| **Secondary infection** | **60/86 (69.8%)** | **74/77 (96.1%)** | **6.606** | **1.58-0.01** | **0.010** |

|  | **Disease severity**  Number of positive/number tested (%) | | **Logistic regression** | | |
| --- | --- | --- | --- | --- | --- |
|  | **DF** | **DHF/DSS** | **Adjusted Odds Ratio** | **95% CI** | ***P* value** |
| Age (number, mean, SD) | 111, 8.72, 9.74 | 87, 7.42, 3.75 | 1.101 | 0.952 - 1.273 | 0.195 |
| DOF 1-3 | 41/90 (45.5) | 14/84 (16.7) | 1 | - | - |
| DOF 4-8 | 49/90 (54.5) | 70/84 (83.3) | 2.102 | 0.829 - 5.329 | 0.118 |
| Log10 cDNA <5 /mL | 33/88 (37.5) | 41/57 (72) | 1 | - | - |
| **Log10 cDNA >5 /mL** | **55/88 (62.5)** | **16/57 (28)** | **0.329** | **0.13 - 0.834** | **0.019** |
| RT-PCR negative | 12/101 (12) | 23/87 (26.5) | 1 | - | - |
| DENV-1 | 38/101 (37.6) | 9/87 (10.3) | 0.579 | 0.102 - 3.276 | 0.536 |
| DENV-2 | 4/101 (4) | 4/87 (4.6) | 1.433 | 0.158 - 12.974 | 0.749 |
| DENV-3 | 42/101 (41.5) | 47/87 (54) | 2.136 | 0.465 - 9.799 | 0.329 |
| DENV-4 | No data | | | | |
| Primary infection | 26/86 (30.2%) | 3/77 (3.9%) | 1 | - | - |
| **Secondary infection** | **60/86 (69.8)** | **74/77 (96.1)** | **6.032** | **1.511 - 24.085** | **0.010** |

* Age, duration after fever onset, NS1 antigen level, DENV serotypes and immune status

SD: standard deviation

CI: confidence interval
